# Effect of monovalency on anti-contactin-1 IgG4

**DOI:** 10.3389/fimmu.2023.1021513

**Published:** 2023-03-14

**Authors:** Guillaume Taieb, Alexandre Jentzer, Elisa Vegezzi, Cinta Lleixà, Isabel Illa, Luis Querol, Jérôme J. Devaux

**Affiliations:** ^1^ Institut de Génomique Fonctionnelle, Université de Montpellier, CNRS, INSERM, Montpellier, France; ^2^ Department of Neurology, CHU Montpellier, Hôpital Gui de Chauliac, Montpellier, France; ^3^ Department of Immunology, CHU Montpellier, Hôpital Saint-Eloi, Montpellier, France; ^4^ Department of Brain and Behavioral Sciences, University of Pavia, Pavia, Italy; ^5^ Neuromuscular Diseases Unit, Hospital de la Santa Creu i Sant Pau, Universitat Autónoma de Barcelona, Barcelona, Spain

**Keywords:** immunoglobulin, demyelination, GBS, auto-immune, myelin, Schwann, axon

## Abstract

**Introduction:**

Autoimmune nodopathies (AN) have been diagnosed in a subset of patients fulfilling criteria for chronic inflammatory demyelinating polyradiculoneuropathy (CIDP) who display no or poor response to intravenous immunoglobulins. Biomarkers of AN are autoantibodies, mainly IgG4, directed against the ternary paranodal complex composed by neurofascin-155, contactin-1 (CNTN1), and Contactin-associated-protein-1 (CASPR1) or against the nodal isoforms of neurofascin. IgG4 can undergo a Fab-arm exchange (FAE) which results in functionally monovalent antibody. This phenomenon differentially affects the pathogenicity of IgG4 depending on the target of autoantibodies. Here, we have evaluated this issue by examining the impact of valency on anti-CNTN1 IgG4 which induces paranodal destruction through a function blocking activity.

**Methods:**

Sera were obtained from 20 patients with AN associated with anti-CNTN1 antibodies. The proportion of monospecific/bispecific anti-CNTN1 antibodies was estimated in each patient by ELISA by examining the ability of serum antibodies to cross-link untagged CNTN1 with biotinylated CNTN1. To determine the impact of monovalency, anti-CNTN1 IgG4 were enzymatically digested into monovalent Fab and tested *in vitro* on cell aggregation assay. Also, intraneural injections were performed to determine whether monovalent Fab and native IgG4 may penetrate paranode, and antibody infiltration was monitored 1- and 3-days post injection.

**Results and discussion:**

We found that the percentage of monospecific antibodies were lower than 5% in 14 out of 20 patients (70%), suggesting that IgG4 have undergone extensive FAE *in situ*. The levels of monospecific antibodies correlated with the titers of anti-CNTN1 antibodies. However, no correlation was found with clinical severity, and patients with low or high percentage of monospecific antibodies similarly showed a severe phenotype. Native anti-CNTN1 IgG4 were shown to inhibit the interaction between cells expressing CNTN1/CASPR1 and cells expressing neurofascin-155 using an *in vitro* aggregation assay. Similarly, monovalent Fab significantly inhibited the interaction between CNTN1/CASPR1 and neurofascin-155. Intraneural injections of Fab and native anti-CNTN1 IgG4 indicated that both mono- and bivalent anti-CNTN1 IgG4 potently penetrated the paranodal regions and completely invaded this region by day 3. Altogether, these data indicate anti-CNTN1 IgG4 are mostly bispecific in patients, and that functionally monovalent anti-CNTN1 antibodies have the pathogenic potency to alter paranode.

## Introduction

A subset of patients with autoimmune neuropathies displays acute or subacute onset, no overt macrophage-mediated demyelination on nerve biopsy, poor response to intravenous immunoglobulins, improvement after Rituximab, and specific autoantibodies directed against the paranodal proteins. Due to these characteristic features, these patients are currently classified under the autoimmune nodopathy diagnostic category (AN) (1). Autoantibodies associated with AN are mainly IgG4 and are directed against the ternary paranodal protein complex composed by neurofascin-155 (Nfasc155), contactin-1 (CNTN1), and Contactin-associated-protein-1 (CASPR1) or against the nodal isoforms of neurofascin (2). Nfasc155 is localized on Schwann cell surface and binds to CNTN1 and CASPR1 that are both localized on the surface of axon in septate-like junction at paranodes. This axoglial junction is important for the clustering/stabilization of voltage-gated sodium channels at the nodes of Ranvier and for myelin insulation (3–7). IgG4 are described as anti-inflammatory isotypes because they are unable to elicit both antibody-dependent cell-mediated cytotoxicity and complement-dependent cell-mediated cytotoxicity (8). However, passive transfer of purified IgG4 from patients with AN reproduces the disease in animals. While anti-Nfasc155 IgG4 promotes a cross-linking of transmembrane Nfasc155 leading to Nfasc155 depletion from the surface cell, anti-CNTN1 IgG4 induces a functional blockade of CNTN1/CASPR1-Nfasc155 interaction (9, 10). The first mechanism interferes on paranode formation and maintenance while the second elicits paranodal destruction. IgG4 is also unique because it exhibits labile disulfide connections between its heavy chains (11). Thus, IgG4 is able to undergo a dynamic swapping leading to half-molecule exchange, also termed Fab arm exchange (FAE) (12). FAE results in bispecific IgG4 that are monovalent to their target. This phenomenon appears to differentially affect the pathogenicity of IgG4 autoantibodies. Indeed, while FAE potentiates the pathogenicity of function blocking anti-Muscle-specific kinase (MuSK) IgG4, we recently demonstrated that FAE reduces the impact of anti-Nfasc155 antibodies which act *via* an antigen-crosslinking mechanism (13–15). Here, we examined whether the valency of anti-CNTN1 IgG4 has a beneficial or detrimental effect.

## Material and methods

### Patients

Sera were obtained from twenty patients with AN associated with anti-CNTN1 IgG4 antibodies, forty-three seronegative CIDP patients seen at CHU Montpellier, and twenty-three healthy donors (Etablissement Français du Sang, Montpellier, France). One sample was obtained from a patient included in the ADHERE trial and was collected prior to treatment. In patients presenting with AN, age, sex, time between sample and disease onset, disability (using the modified Rankin scale [mRS] and the Overall Neuropathy Limitation Scale [ONLS]), nerve conductions studies (according to the electrodiagnostic criteria of European Academy of Neurology/Peripheral Nerve Society guideline) (1), and the presence of nephrotic syndrome were assessed. Titers of anti-CNTN1 and isotype were determined in each patient by ELISA. Titers were defined as the greatest serum dilution resulting in a positive ELISA test. Written informed consent were obtained from all patients.

### Antibody purification and cleavage

IgG1, IgG3 and IgG4 fractions were purified with CaptureSelect™ affinity matrix (191303005, 191304005, 2942902005, ThermoFisher scientific, Waltham, MA) on an AKTA Go (Cytiva, Marlborough, MA). Antibodies were desalted to artificial cerebrospinal fluid using HiTrap Desalting column (GE17-1408-01, Cytiva), concentrated at 10 mg/ml, filter sterilized and stored at -20°C until use. Fab fragments were generated using Pierce’s Fab preparation kit (44985, Thermofisher) according to manufacturer’s protocols. Fab fragments were dialyzed to artificial cerebrospinal fluid, concentrated at 10 mg/ml, and filter sterilized. Digestion was monitored by migration on 4-20% SDS-PAGE gels (**Supplementary Figure 1**).

### Monospecificity ELISA assay

Recombinant human His-tagged CNTN1 (Met1-Ala993) was produced in HEK293T cells and purified with HisTrap HP column (GE17-5247-01, Cytiva), then was biotinylated as previously described (14). MaxiSorp microtiter plates were coated overnight with 25 ng of untagged human CNTN1 at 4°C. Wells were blocked with 0.5% casein sodium 0.05% Tween 20 in PBS for 1 hour at 37°C, then incubated overnight at 4°C with patients’ sera diluted 1:10 or with increasing concentration of purified IgG3 anti CNTN1 (ranging from 50 ng to 20 µg). The day after, wells were incubated with 25 ng of biotinylated-CNTN1 for 1 hour at 37°C, and the reactivity was revealed using peroxidase-conjugated streptavidin (1:2000; 18-152, Merck-Millipore, Burlington, MA) and SIGMAFAST OPD (P9187, Merck-Millipore). The percentage of monospecific antibodies was interpolated from the calibration curve obtained with anti-CNTN1 IgG3 as detailed in **Supplementary Method** and **Supplementary Figure 2**. The percentage of monospecific anti-Nfasc155 antibodies was measured in a similar manner against 50 ng of untagged human Nfasc155 as described in **Supplementary Method**.

### Cell aggregation assay

HEK cells were plated in 6-well plates at a density of 500,000 cells/wells and transiently transfected using JetPEI (POL101-10N, Polyplus-transfection, Illkirch-Graffenstaden, France) with mcherry-conjugated (red) rat Nfasc155 or with both rat CNTN1 and GFP-conjugated (green) rat CASPR1, or with only peGFP-N1. The day after, cells were trypsinized using 0.25% trypsin in PBS and suspended in 1 mL of serum free Opti-MEM medium (11564506, ThermoFisher Scientific). Cells were then mixed together in a 1:1 ratio (400,000 cells/ml) in presence of 10 µg of purified antibodies (including either control IgG4 from healthy donors, native anti-CNTN1 IgG4, or monovalent Fab-reactive to CNTN1 from patients AN1 and AN2) and agitated at 100 rpm for 2 hours at 37°C. Fifty microliters of cell suspension were then mounted between slides and coverslips, and immediately observed using an ApoTome fluorescence microscope at the 10X objective. Aggregates were defined as clusters of cells of at least 4 cells. Aggregates showing interactions between green and red cells were considered as cell clusters with contacts. In addition, the percentage of green cells was quantified in each cell clusters. Four experiments were performed for each condition, and a minimum of 40 cell clusters were quantified per experiments.

### Intraneural injections and quantification of antibody infiltration

Four adult Wistar rats (9 weeks old) were anesthetized with Isovet and received a subcutaneous injection of buprenorphine for pain relief. Since native IgG4 and Fab do not have the same molecular mass (150 kDa vs 50 kDa), 1 μl of native anti-CNTN1 IgG4 at 10 μg/μl or 1 µl anti-CNTN1 Fab at 3 μg/μl from patient AN1 were injected with a glass micropipette in the sciatic nerves at the level of the sciatic notch.

One or three days after surgery, injected nerves were dissected out, fixed in 2% paraformaldehyde in PBS for 1 hour at 4°C, then rinsed in PBS. Axons were gently teased, dried on glass slides, and stored at -20°C. Teased fibers were permeabilized by immersion in -20°C acetone for 10 min, blocked at RT for 1 hour with PBS containing 5% fish skin gelatin and 0.1% Triton X-100, then incubated overnight at 4°C with a goat antibody against CNTN1 (1:2000; AF904, R&D Systems, Mineapolis, MN). The slides were then washed several times and incubated with the following conjugated secondary antibodies (Jackson ImmunoResearch, West Grove, PA) diluted 1:500 in blocking solution: donkey anti-human IgG Alexa 488 (709-545-149) and donkey anti-goat Alexa 594 (705-585-147). Slides were mounted with Mowiol plus 2% DABCO, and examined using an ApoTome fluorescence microscope (ApoTome, AxioObserver and AxioCam MRm, Carl Zeiss MicroImaging GmbH). Antibody infiltration was quantified using ImageJ software (NIH). The length of IgG labeling (i.e. native anti-CNTN1 IgG4, monovalent Fab-reactive to CNTN1, or control IgG4) and the length of CNTN1 labeling were measured, then the ratio IgG length/paranode length was calculated. Digital images were manipulated into figures with CorelDraw and Corel Photo-Paint (Corel Corporation, Ottawa, Canada).

### Statistics

Statistical significance was assessed by unpaired and paired two-tailed Student’s t tests, Kolmogorov-Smirnov tests, or by one-way ANOVA followed by Bonferroni’s *post-hoc* tests using GraphPad Prism (GraphPad Software, San Diego, CA). Linear regression and Spearman correlation were performed using GraphPad Prism. P values inferior to 0.05 were considered significant.

### Standard protocol approvals, registrations, and patient consents

The study was approved by the Ethics Committee of Montpellier University Hospital (IRB-MTP-2020-01-20200339). All animal experiments were in lines with the European community’s guiding principles on the care and use of animals (2010/63/EU) and were approved by the local ethical committee and by the “ministére de l’éducation nationale de l’enseignement supérieur et de la recherche” (APAFIS#3847-2016012610089856v5). Experiments were performed without blinding.

## Results

### Characteristics of patients with AN related to anti-CNTN1 IgG4

Twenty patients (19 males and 1 female) with AN associated with anti-CNTN1 IgG4 antibodies were enrolled. The median age of onset was 64 (IQR 55-75). Median values on disability scales were 4.5 (IQR 4-5) on mRS and 9.5 (IQR 8-12) on ONLS. According to the electrodiagnostic criteria of the European Academy of Neurology/Peripheral Nerve Society, motor nerve conduction studies showed demyelinating features in all patients (i.e. strongly supportive of demyelination). Nephrotic syndrome was present in 9 patients. The median anti-CNTN1 titers was 6200 (IQR 2700-8400) and isotypes were IgG4 in all patients; additionally, IgG3 was also detected in five, IgG1 in three, and IgG2 in one patient (**Table 1**).

**Table 1:** Antibody titers and clinical features of CNTN1-reactive patients.

	Titers 1/x	Isotype	Monospecific anti-CNTN1 (%)	Gender	Age	Clinical score		Nephropathy	Response to treatment
						mRS	ONLS		
AN1	52700	IgG4,3,1	24,9	M	71	5	12	+	Plex +, Ritux +
AN2	20000	IgG4	5,1	M	59	NA	8	–	IVIg -
AN3	500	IgG4	0,1	M	39	5	12	–	IVIg -, Cx +
AN4	5000	IgG4	3,1	M	70	NA	8	+	IVIg -, Cx partial
AN5	4200	IgG4	1,5	M	75	5	9	+	IVIg -, Ritux +
AN6	6200	IgG4	3,2	M	58	4	4	+	NA
AN7	7500	IgG4	0,3	M	60	4	9	–	IVIg -, Cx -
AN8	1200	IgG4	0,6	M	79	5	12	–	IVIg -, Cx -
AN9	6500	IgG4,3	8,6	M	80	5	12	+	IVIg -, Cx -
AN10	2700	IgG4	1,9	F	40	2	3	–	IVIg -, Cx +
AN11	1900	IgG4,3	0,1	M	54	5	12	–	IVIg +, Cx -
AN12	2500	IgG4	0,7	M	66	5	10	+	IVIg -, Cx -
AN13	7000	IgG4,3,2,1	21,5	M	74	4	10	–	IVIg -, Cx -
AN14	8400	IgG4	0,3	M	37	NA	8	+	IVIg -, Cx +
AN15	7000	IgG4	0,2	M	52	4	6	–	Cx -
AN16	4400	IgG4	0,7	M	82	NA	10	–	IVIg -, Cx -
AN17	32000	IgG4	18,9	M	75	4	NA	–	IVIg -
AN18	900	IgG4,1	0,7	M	61	4	NA	–	Cx +, Aza +
AN19	16000	IgG4,3	11,3	M	71	5	NA	+	Cx -, IVIg -, PLEX +
AN20	2900	IgG4	0,3	M	62	4	NA	+	Cx-, IVIg –, Aza -, PLEX + RTX + Rituximab

Aza, azathioprine; Cx, corticosteroids; F, Female; IVIg, Intravenous Immunoglobulin; M, Male; mRS, modified Rankin Score; NA, not available; ONLS, Overall Neuropathy Limitation Score; Plex, Plasma Exchange; Ritux, Rituximab. In the nephropathy column, +/- indicate the presence or absence of nephrophathy, respectively. In the response to treatment column, +/- indicate the responsiveness or lack of response to treatment, respectively.

### Anti-CNTN1 IgG4 have majorly undergone FAE *in situ*

**Figure 1:**
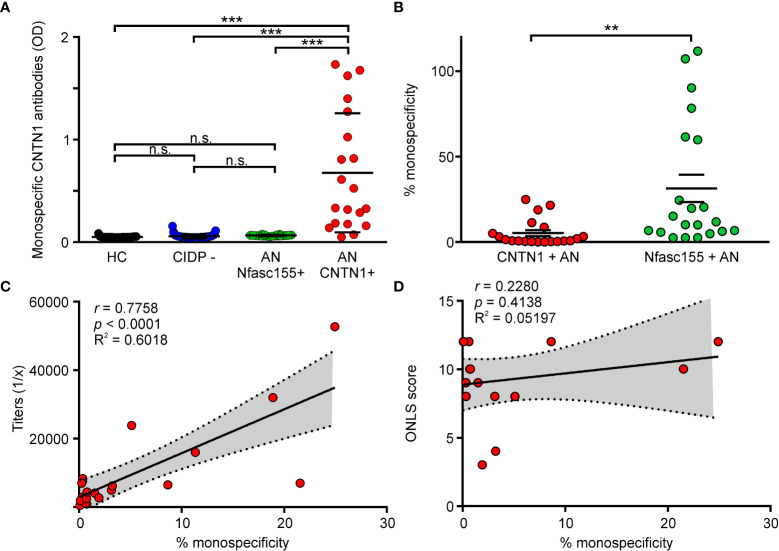
Fab-arm exchange occurs in patient with AN. **(A)** The capacity of serum antibodies to cross-link untagged CNTN1 with biotinylated CNTN1 was measured by sandwich ELISA in healthy controls (HC; n = 23), seronegative CIDP patients (CIDP -; n = 43), Nfasc155+ autoimmune nodopathy (AN Nfasc155 +; n = 21) and CNTN1+ autoimmune nodopathy (AN CNTN1+; n = 20). Antibodies from HC, seronegative patients or Nfasc155+ did not cross-link CNTN1. By contrast, CNTN1+ AN patients significantly cross-linked CNTN1 (*** *P*<0.001 by one-way ANOVA followed by Bonferroni’s *post-hoc* tests). **(B)** The percentage of monospecific antibodies was calculated by interpolating the data from a calibration curve obtained with anti-CNTN1 IgG3. The percentage of monospecific anti-CNTN1 antibodies in CNTN1+ AN (n = 20) was significantly smaller compared to the percentage of monospecific anti-Nfasc155 antibodies in Nfasc155+ AN (n = 21) (** *P*<0.005 by unpaired two-tailed Student’s t tests). **(C)** The titers of anti-CNTN1 IgG4 correlated with the percentage of monospecific CNTN1 antibodies calculated in each patient. **(D)** The clinical severity (ONLS) is plotted against the percentage of monospecific antibodies in each patient (n = 16). No significant correlation was found. P value, Spearman’s correlation coefficient (r), R square (R^2^) and 95% confidence band (dotted lines) are indicated on the graph. n.s.: non-significant, ONLS: Overall Neuropathy Limitation Score.

To determine whether anti-CNTN1 antibodies are predominantly mono- or bispecific *in situ*, we have examined the potency of anti-CNTN1 autoantibodies to cross-link untagged CNTN1 with biotinylated CNTN1. For that purpose, sera from 20 CNTN1+ AN patients were tested by sandwich-ELISA, as well as the sera from 23 healthy donors, 21 Nfasc155+ AN patients, and 43 seronegative CIDP patients (**Figure 1**). Those patients were tested for antibodies against biotin, and none were positive (**Supplementary Figure 4**). Anti-CNTN1 autoantibodies from AN patients were found to significantly cross-link CNTN1 with biotinylated CNTN1, albeit with variable degrees among the cohort. By contrast, healthy donors Nfasc155+ AN, and seronegative CIDP patients did not bind CNTN1. The percentages of monospecific antibodies were then calculated by interpolating the data to a standard curve obtained with increasing concentration of purified anti-CNTN1 IgG3 (**Supplementary Material**, **Supplementary Figure 2**). For comparison, the percentages of monospecific anti-Nfasc155 antibodies were calculated in a cohort of 21 Nfasc155+ AN patients. Sixteen out of the 20 CNTN1+ AN patients presented with less than 10% of monospecific anti-CNTN1 antibodies (**Figure 1B**). The mean percentage of monospecific anti-CNTN1 antibodies was 5.2 +/- 7.8, and was strongly inferior to that of monospecific anti-Nfasc155 antibodies in the Nfasc155+ cohort (31.4 +/- 36.9). In a preliminary study, the percentage of monospecific antibodies was also evaluated in the purified IgG4 fraction from 4 CNTN1+ AN patients (**Supplementary Figure 3**). In those patients, the percentage of monospecific anti-CNTN1 antibodies calculated in the IgG4 fraction were lower than those detected in serum. This further indicated that the majority of anti-CNTN1 IgG4 have undergone FAE *in situ*, and are less likely to cross-link their targets compared to anti-Nfasc155 IgG4.

We then investigated whether the levels of monospecific antibodies influenced clinical severity. The levels of monospecific anti-CNTN1 antibodies strongly correlated with antibody titers in AN patients (**Figure 1C**; *p* < 0.001). However, no correlation was found between the amount of monospecific anti-CNTN1 antibodies and clinical severity (**Figure 1D**; *p* = 0.3322). Most patients presented with a severe clinical presentation (**Table 1**; mean ONLS score = 9 +/- 3) irrespectively of the levels of monospecific antibodies. No significant correlation was detected between the levels of monospecific antibodies and any clinical features.

### Monovalent anti-CNTN1 IgG4 blocks CNTN1/CASPR1-Nfasc155 interaction

**Figure 2:**
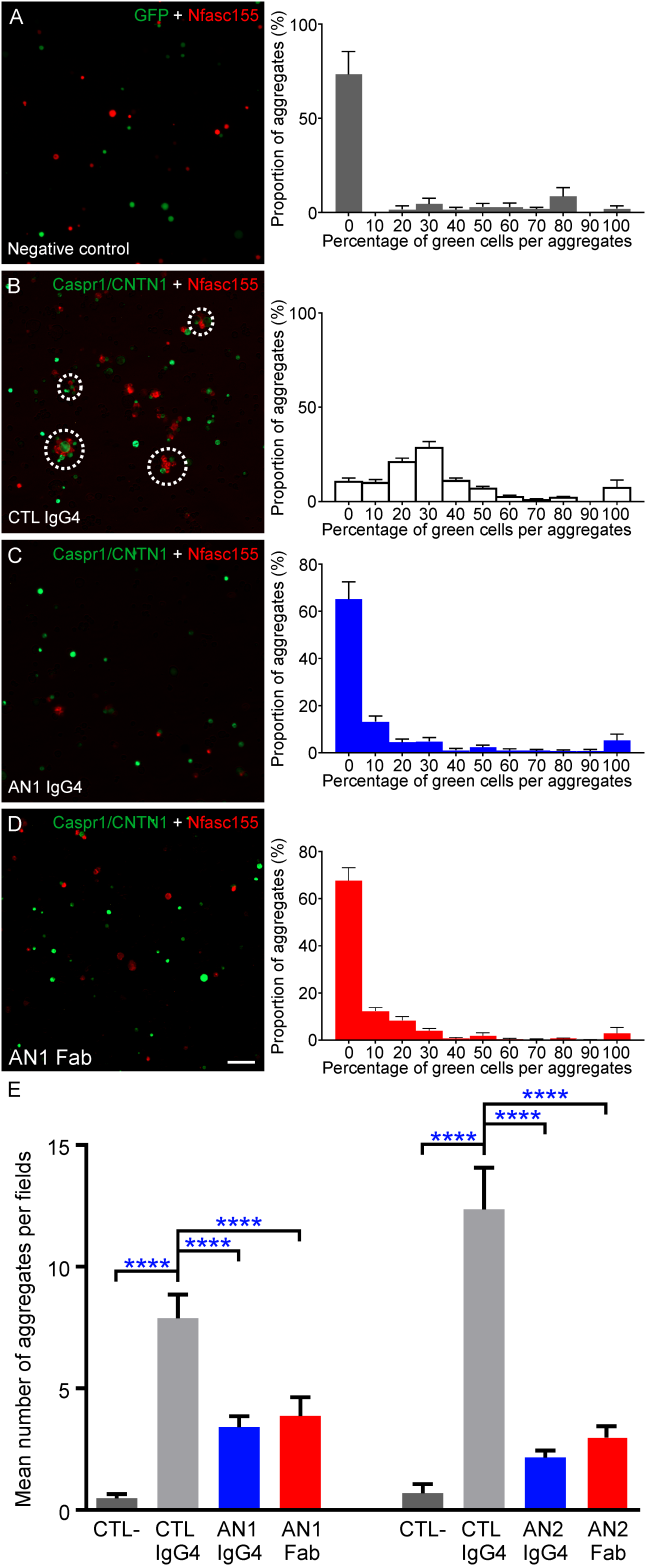
Monovalent Fab are sufficient to inhibit the interaction between CNTN1/CASPR1 and Nfasc155. **(A-D)** These are representative images of cell aggregation assays. HEK293T cells were transfected with CASPR1-GFP/CNTN1 (green) and were incubated with cells expressing mcherry tagged Nfasc155 (red) in presence of control IgG4 from a healthy donor (CTL; B), native CNTN1-reactive IgG4 from patient AN1 **(C)** or monovalent Fab-reactive to CNTN1 **(D)**. As negative controls, cells expressing GFP (green) were incubated with cells expressing mcherry tagged Nfasc155 **(A)**. The right panels represent the distribution of the percentage of green cells per aggregate in each aggregate (N = 4 distinct experiments for each condition). **(E)** The native IgG4 and monovalent Fab were tested from two AN patients reactive against CNTN1 (AN1 and AN2). The number of cell aggregates per visualization field was counted in ten images and averaged (N = 4 distinct experiments per condition). Both native IgG4 and monovalent Fab reactive against CNTN1 significantly inhibited the interaction between CASPR1/CNTN1 and Nfasc155 (**** *P*<0.0001 by one-way ANOVA followed by Bonferroni’s *post-hoc* tests). No significant differences were observed between the effect of monovalent or native IgG4 for both patients. Native anti-CNTN1 IgG4 do not promote CNTN1/CASPR1 antigen clustering **(C)**. Bars represent mean +/- S.E.M. Scale bar: 10 µm.

Previous studies have shown that anti-CNTN1 IgG4 have a function blocking activity and block the interaction between CNTN1/CASPR1 and Nfasc155. In order to determine whether the valency of anti-CNTN1 influence its function blocking activity, the IgG4 from two CNTN1+ AN patients were purified and reduced into monovalent Fab by enzymatic cleavage. These patients presented with different percentages of monospecific antibodies of 24.9% (AN1) and 5.1% (AN2). The potency of native IgG4 or Fab fragment to inhibit the interaction between CNTN1/CASPR1 and Nfasc155 was tested using a cell aggregation assay. HEK cells transfected with CNTN1 and CASPR1-GFP were incubated for 2 hours with HEK cells transfected with mcherry-Nfasc155, then visualized under a microscope. The number of cell aggregates including green and red cells was then quantified in each visualization field (n = 4 experiments per condition, 10 visualization fields were examined by experiments). As negative controls, Nfasc155-transfected cells were incubated with GFP-transfected cells. In control condition, Nfasc155-transfected cells readily aggregated with CNTN1/CASPR1-transfected cells and an average of 8 to 12 aggregates were found in each visualization field (**Figure 2**). These aggregates were composed of nearly equal number of green and red cells (**Figure 2B**). The adjunction of native anti-CNTN1 IgG4 from both AN1 and AN2 strongly inhibited the capacity of Nfasc155-transfected cells to aggregates with CNTN1/CASPR1-transfected cells (*P*<0.0001 by one-way ANOVA followed by Bonferroni’s *post-hoc* tests), and did not promote CNTN1/CASPR1-transfected cell clustering.

Monovalent Fab fragments induced similar effects and potently abrogated the interaction between Nfasc155 and CNTN1/CASPR1 *in vitro*. No significant difference was seen between the effect of native or monovalent anti-CNTN1 IgG4 (*P*>0.05 by one-way ANOVA followed by Bonferroni’s *post-hoc* tests). As most IgG4 appears to have undergone FAE in CNTN1+ AN patients, these results suggested that bispecific (functionally monovalent) anti-CNTN1 IgG4 blocks CNTN1 function.

### Anti-CNTN1 fab penetrates paranodal regions

**Figure 3:**
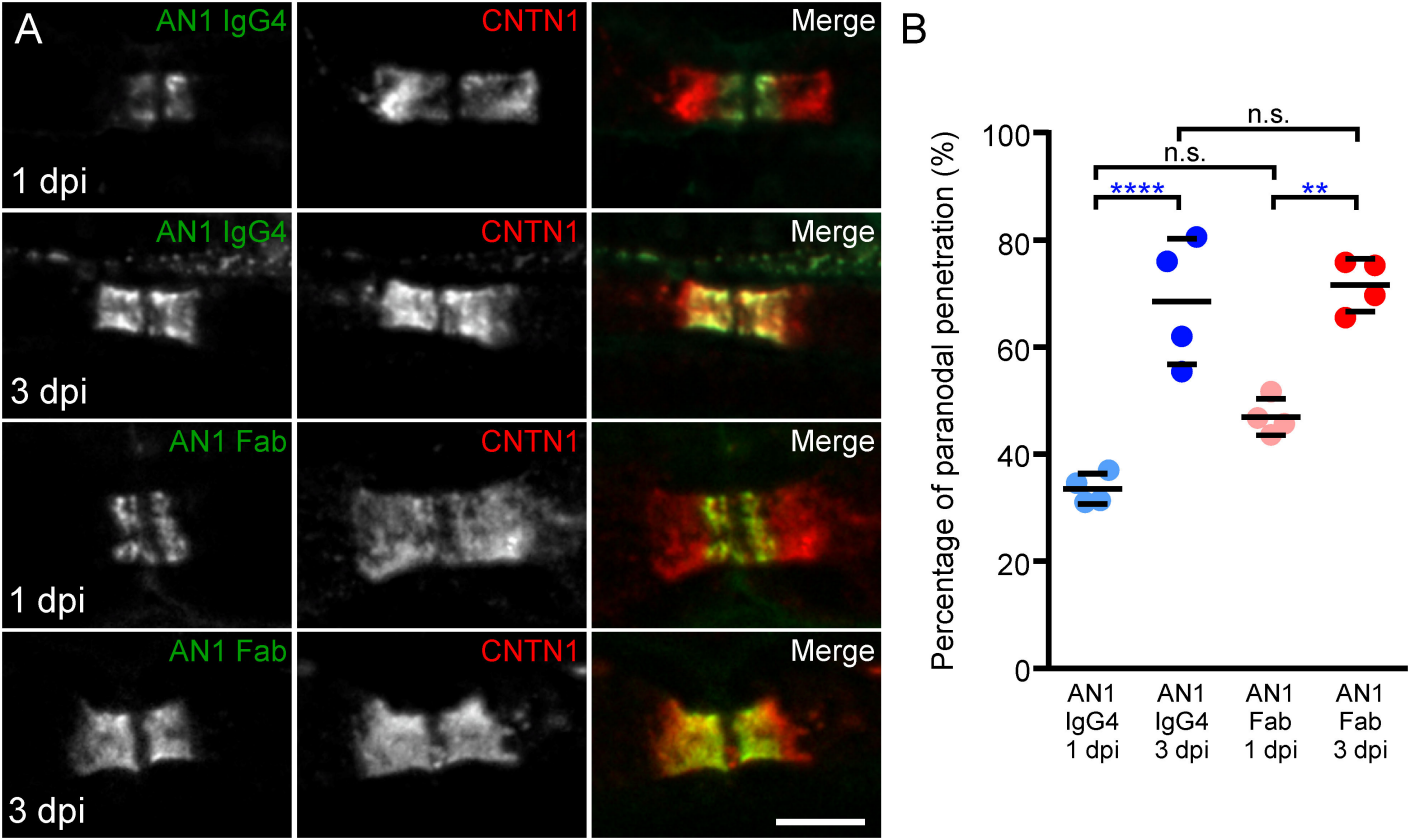
Mono- and bivalent IgG4 infiltrate paranodal regions. **(A)** These are teased sciatic nerve fibers from adult Lewis rats which have received a single intraneural injection of native CNTN1 reactive IgG4 (top panels; n = 4 at each time point) or monovalent Fab (lower panels; n = 4 at each time point). Nerves have been collected 1- or 3-days post-injection (dpi) and have been stained for CNTN1 (red) and IgG (green). **(B)** The percentage of IgG infiltration was measured in each animal at 1 and 3 dpi, and the mean percentage of infiltration was calculated (n = 4 for each condition). The paranodal infiltration of native IgG4 reactive to CNTN1 was significantly higher at 3 dpi compared to 1 dpi (**** *P*<0.0001, by one-way ANOVA followed by Bonferroni’s *post-hoc* tests). The percentage of infiltration of monovalent Fab was also significantly higher at 3 dpi compared to 1 dpi (** *P*<0.01 by one-way ANOVA followed by Bonferroni’s *post-hoc* tests). No significant difference was seen between monovalent Fab and native IgG4 infiltration at both times. Bars represent mean and S.D. Scale bar: 5 µm.

Because Fab fragments inhibit CNTN1/CASPR1-Nfasc155 interaction, we next investigated whether the reduction of anti-CNTN1 IgG4 into monovalent Fab alters its potency to invade the paranodal regions. For that purpose, intraneural injections of native anti-CNTN1 IgG4 or Fab fragments from patient AN1 were performed in the sciatic nerves of adult rats. In order to inject equimolar amounts of antibodies (Fab fragment being three time smaller than native IgG), 10 µg of native IgG4 and 3 µg of Fab were injected. The penetration of antibodies within the paranodal region was monitored one- and three-days post-injection (**Figure 3**; **Supplementary Figure 5**). As previously described, by one day post-injection, anti-CNTN1 IgG4 were detected within the paranodal regions at the border of the nodes of Ranvier, then progressively invaded the entire paranode by three days (**Figure 3**; **Supplementary Figure 5**). Monovalent Fab fragment also readily penetrated the paranodes *in vivo* by one day. Fab fragment of anti-Nfasc155 IgG4 or unreactive IgG4 do not infiltrate paranodal regions (14). It thus seems unlikely that Fab infiltration is solely due to the small size of Fab and to unspecific diffusion across the paranode. At three days post-injection, the extent of diffusion of anti-CNTN1 Fab or IgG4 across the paranodes was significantly higher than that at one-day post injection (*P*<0.0001 and *P*<0.01, respectively by one-way ANOVA followed by Bonferroni’s *post-hoc* tests). No significant difference was seen between monovalent Fab and native IgG4 at both time points, indicating that monovalent anti-CNTN1 Fab antibodies have the same capacity to invade paranode as native antibodies.

## Discussion

In this study, the levels of mono/bispecific anti-CNTN1 IgG4 were estimated in AN patients as well as the pathogenic function of monovalent anti-CNTN1 IgG4. Our results suggest that most anti-CNTN1 IgG4 have already undergone FAE *in situ* since only 5% of these were bivalent and monospecific against CNTN1. In addition, the proteolytic digestion of these antibodies into monovalent Fab did not alter their function and monovalent Fab potently inhibited the interaction between CNTN1/CASPR1 and Nfasc155, and infiltrated paranodal regions. This suggested that bispecific antibodies may alter paranodal complex association.

IgG4 are known to undergo FAE *in situ* and to coexist as functionally monovalent antibodies. This property is central for the physiological non-inflammatory function of IgG4. In addition, it appears to influence the pathogenicity of IgG4 autoantibodies in autoimmune diseases. In myasthenia gravis (MG) associated with anti-MuSK IgG4, FAE enhances the pathogenic function of these autoantibodies by generating monovalent bispecific antibodies unable to cluster MuSK but capable of blocking the interaction between MuSK and Low-density lipoprotein receptor related protein 4 (Lrp4) (15). In MuSK+ MG patients, circulating autoantibodies were reported to be majorly bispecific. By contrast, in Nfasc155+ AN, FAE was reported to have an opposite effect, and to decrease the potency of anti-Nfasc155 IgG4 to cluster Nfasc155 and to block paranode formation (14). Here and in a previous study, we found that 21-31% of the anti-Nfasc155 IgG4 were monospecific and that some patients showed nearly 100% monospecific antibodies. The reasons why the levels of bivalent monospecific anti-Nfasc155 IgG4 is high is uncertain, but could be due to a selection bias. Indeed, these autoantibodies were only tested when the neuropathic condition was declared or severe, thus when high levels of pathogenic monospecific bivalent anti-Nfasc155 IgG4 are circulating. Conversely, in anti-CNTN1+ AN, the proportion of monospecific antibodies was low (~ 5%) and most antibodies appeared bispecific. Like in anti-MuSK+ MG, IgG4 had a function blocking activity and monovalent Fab-reactive to CNTN1 could potently block the interaction between CNTN1/CASPR1-Nfasc155. As indicated in the **Supplementary Material**, one of the limitations of the study is that the percentage of monospecific antibodies were measured in patients’ sera and may have been overestimated due to the coexistence of IgG1 and IgG3. The percentage of monospecific IgG4 in CNTN1+ AN patients may thus be lower.

Our results suggest that the impact of valency on pathogenicity strongly depend on the mode of action of the IgG4: function blocking or antigen-crosslinking mechanism. When IgG4 have a function blocking activity, monovalent antibodies can participate to the pathogenic mechanism. Conversely, if IgG4 act through an antigen clustering, then monovalency reduces the pathogenic mechanism, probably because antigen-crosslinking requires a monospecific and bivalent antibody. These differences may explain why patients with anti-CNTN1 IgG4 have a more severe presentation. While FAE naturally dampen the pathogenicity of anti-Nfasc155 IgG4, it may not preclude the action of anti-CNTN1 IgG4. However, this cannot be formally ascertained here, albeit our results suggest this pathophysiological mechanism. Indeed, the pathogenic action of pure monospecific bivalent anti-CNTN1 IgG4 has not been specifically investigated, neither the impact of FAE. Of course, this study has several limitations. The levels of monospecific IgG have been inferred from patients’ sera and not from purified IgG4, thus the presence of other IgG isotypes may have led to an overestimation of the percentage of monospecificity. Also, those levels were interpolated from a calibration curve obtained with anti-CNTN1 IgG3, and thus are rough estimate rather than definite levels.

Although the inhibition of CNTN1 function seems to be the main pathogenic process leading to paranode alteration, antigen-crosslinking mechanism may also exist in other regions *in situ*. Besides paranodes, CNTN1 is also present on the surface of dorsal root ganglia and on podocytes, which may explain sensory ataxia and nephrotic syndrome, respectively. Recently, Gruner and colleagues showed that anti-CNTN1 IgG1-3 and F(ab’)_2_ fragment decreased CNTN1 surface expression on dorsal root ganglia. As anti-CNTN1 Fab fragment abolished this effect, a cross-linking mechanism and subsequent internalization of CNTN1 have been suggested (16). It still remains to be demonstrated whether monospecific, and not bispecific, anti-CNTN1 IgG4 promote the same effect. In our study, 9 patients also displayed a nephrotic syndrome. Although CNTN1 is expressed by podocytes in normal kidney and is the target of anti-CNTN1 IgG4 in patients with nephrotic syndrome, it remains unknown whether the pathogenic mechanism in membranous nephropathy results from a function blocking, antigen-crosslinking or immune-complex deposition (17). Here, we did not find a correlation between the presence of a nephrotic syndrome and the percentage of circulating monospecific antibodies. However, we cannot exclude that IgG4 deposits in kidney are due to bivalent monospecific autoantibodies.

Immunotherapies classically used in autoimmune neuropathies, such as intravenous immunoglobulins, are usually ineffective in AN that, conversely, respond very well to B-cell depleting therapies such as Rituximab (1). However, axonal loss is sometimes already advanced before Rituximab fully exerts its effect. Because IgG4 has poor Fc-mediated effector functions, imlifidase (a therapeutic enzyme that cleaves Fc domain of IgG) and anti-complement therapy may be disregarded as useful options in anti-CNTN1+ AN. Finally, neonatal Fc receptor targeting agents may be a promising therapy for AN since this treatment seems to be effective in mouse model for IgG4 MuSK+ MG (18).

Our study underscores that the paranodopathy mechanism in anti-CNTN1+ AN is by the blockade of the CNTN1/CASPR1-Nfasc155 interactions by anti-CNTN1 antibodies and that anti-CNTN1 Fab fragments or bispecific antibodies can carry a function blocking activity. This may explain why patients with anti-CNTN1 IgG4 which have undergone extensive FAE still present a severe phenotype.

## Data availability statement

The raw data supporting the conclusions of this article will be made available by the authors, without undue reservation.

## Author contributions

All authors drafted and revised the manuscript. All authors were involved in the acquisition of data, analysis and interpretation of data. All authors contributed to the article and approved the submitted version.
