# Supplementary material for: Effect of monovalency on anti-contactin-1 IgG4
Source: Front Immunol. 2023 Mar 14;14:1021513. doi: 10.3389/fimmu.2023.1021513 (PMC10045471; doi:10.3389/fimmu.2023.1021513)
Supplement: Supplementary file 1 [file DataSheet_1.docx]

Supplementary Material

Supplementary method

Monospecificity ELISA assay – setting and calculation method

Step 1 - MaxiSorp microtiter plates were coated overnight with 25 ng of untagged human CNTN1 at 4°C as for routine ELISA, then blocked with 100 µl of 0.5% casein sodium 0.05% Tween 20 in PBS for 1 hour at 37°C.

Step 2 - wells were incubated overnight at 4°C with increasing quantities (ranging from 50 ng to 20 µg) of purified IgG3 from an anti-CNTN1 patient (patient AN1), purified IgG1 from an anti-CNTN1 patient (patient AN1), or purified IgG4 fractions from four AN patients (AN1, AN2, AN4 and AN19) in blocking solution.

Step 3 - The day after, wells were washed six times and incubated with 25 ng of biotinylated-CNTN1 for 1 hour at 37°C.

Step 4 - The wells were then washed six times, and incubated for 1 hour with peroxidase-conjugated streptavidin (1:2000) in blocking solution.

Step 5 - The wells were then washed six times, then revealed for 15 min at RT with 50 µl SIGMAFAST OPD diluted in water and the reaction was stopped with 50 µl of 2N HCl. The optical density was read at 490 nm.

As shown in Supplementary figure 2B, for IgG3 or IgG1 fractions reactive against CNTN1, the OD values increased as a function of antibody quantity reaching a plateau at 10 and 20 µg, and were fitted with a nonlinear regression using one site specific binding with Hill slope equation. At high concentrations, it thus appeared that every antigenic site in the wells were occupied by an antibody (Supplementary figure 1A). The maximal OD values were obtained with IgG3 or IgG1 fractions reactive against CNTN1 for quantities around 10 µg. Since both antibodies are bivalent monospecific, this OD thus corresponded to a 100% occupancy of antigenic sites by monospecific antibodies. The binding of IgG4 to CNTN1 also increased as a function of antibody amount and tended to a plateau for the same antibody amounts (10-20 µg), but reached lower maximal OD values compared to purified IgG1 or IgG3. Also, the maximal plateau value varied between patients. This indicated that IgG4 tend to occupy all antigenic sites, however, since those antibodies are a blend of mono- and bispecific antibodies, the maximal OD value was lower and was limited by the proportion of monospecific IgG4 in the sample (as depicted in the scheme in Supplementary figure 2A).

Since IgG1 and IgG3 are 100% monospecific, the monospecificity-quantity curve can be assimilated to a calibration curve to estimate the amount (in µg) of monospecific antibodies present in 10 µg of IgG4, and thereby the percentage of monospecific IgG (see examples in Supplementary figure 2D). To determine which calibration curves (IgG1 or IgG3) suit better to quantify monospecific antibody percentages, the curves obtained with IgG1 and IgG3 were normalized to the maximal OD values at 20 µg and were compared to the mean normalized curve of the four AN patients’ IgG4 (Supplemental figure 2C). The binding profile of anti-CNTN1 IgG4 matched more closely to that of anti-CNTN1 IgG3, and this latter curve was used to interpolate the percentage of monospecific antibodies.

To validate the quantification method, blends of mono- and bispecific antibodies were artificially created by mixing native IgG3 reactive against CNTN1 with monovalent Fab fragments (generated from purified anti-CNTN1 IgG3) in 1:1 and 1:2 ratios to obtain blends with 50% or 33.33% of monospecific IgG, respectively. As for IgG4, the monospecificity-quantity curves obtained with these blends reached lower maximal OD values compared to pure IgG3 (Supplementary figure 2D). The OD values obtained with a quantity of 10 µg of IgG3/Fab mixture were interpolated to the anti-CNTN1 IgG3 calibration curve to calculate the corresponding quantities of monospecific antibodies, and thereby the percentage of monospecific IgG. As expected, 1:1 IgG3/Fab mixture reduced the levels of monospecific IgG to 44.6 % (expected 50%), and 1:2 IgG3/Fab mixture to 31.9% (expected 33.33%), thus validating the quantification method.

This methodology was then used to quantify the percentage of monospecific IgG4 in the four AN samples. It was found that these patients presented a low percentage of monospecific IgG4 ranging between 1 to 11 % (Supplementary table 1). Because the amount of serum/plasma required to purify and test IgG4 were important and were limiting for the study, we decided to develop a similar assay using crude serum. In a first manner, the sera from patients AN1, 2, 4 and 19 were used to implement the test and for comparison. The sera were tested using the same method as described above with the difference that in step 2 the wells were incubated overnight at 4°C with increasing serum concentrations (ranging from 1/1000 to 1/5) in blocking solution. As for purified antibodies, the monospecificity-concentration curves increased as a function of antibody quantity, were fitted with a nonlinear regression using one site specific binding with Hill slope equation, and reached a plateau for a dilution of 1/10 (Supplementary figure 2E). This indicated that at this dilution, every antigenic site was occupied by an antibody, and this dilution was assumed to be equal to the binding of 10 µg of antibodies. As above, the OD values obtained at this dilution were used to interpolate monospecific antibody percentages in each sample (Supplementary table 1). Comparison with purified IgG4 indicated that values measured in sera matched closely to those found in purified IgG4. An overestimation of the percentage of monospecific antibodies was nonetheless evident in sera compared to purified IgG4. This overestimation was detected in all four patients regardless of the presence of IgG1 or IgG3 against CNTN1 (Supplementary table 1).

To further evaluate whether monospecificity percentages calculated from sera reflect those calculated in IgG4 fractions, a comparison was performed between sera and purified IgG4 from Nfasc155+ AN patients (Supplementary figure 3). The percentage of monospecific anti-Nfasc155 IgG was calculated using the method described above with the difference that plates were coated with 50 ng of untagged human Nfasc155 in step 1 and were incubated with 50 ng of biotinylated-Nfasc155 in step 3. Purified anti-Nfasc155 IgG1 were used for calibration curve. As depicted in Supplementary figure 3, a strong correlation was found between the percentage of monospecificity calculated from crude serum and purified IgG4 in Nfasc155+ AN. The percentage of monospecificity calculated in crude serum were very close to those found in purified IgG4, presumably because of the lower prevalence of IgG1 and IgG3 in Nfasc155+ AN patients’ sera. One limitation of this method thus seems to be that it may overestimate the percentage of monospecific antibodies in CNTN1+ AN patients’ sera.

Supplementary tables

**Supplementary table 1: Percentage of monospecific anti-CNTN1 antibodies in purified IgG4 and serum.**

|  | Isotype | Percentage of monospecific anti-CNTN1 in serum | Percentage of monospecific anti-CNTN1 in purified IgG4 |
| --- | --- | --- | --- |
|  |  |  |  |
| AN1 | IgG4,3,1 | 24,9 | 10.9 |
| AN2 | IgG4 | 5,1 | 1.4 |
| AN4 | IgG4 | 3,1 | 1.2 |
| AN19 | IgG4,3 | 11,3 | 8.7 |

## Supplementary Figures


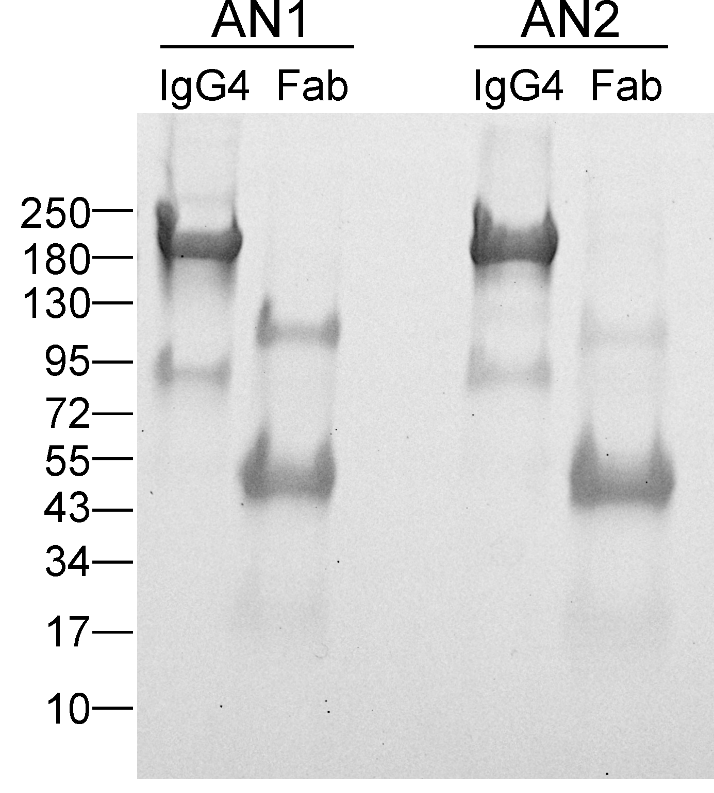


**Supplementary Figure 1** Digestion of IgG4 into Fab fragment. 10 µg of native IgG4 or Fab fragment from patients AN1 and AN2 were separated on 4-20% SDS-PAGE gels and stained with Coomassie blue to ensure proper digestion. Molecular weight markers are shown on the right in kDalton.


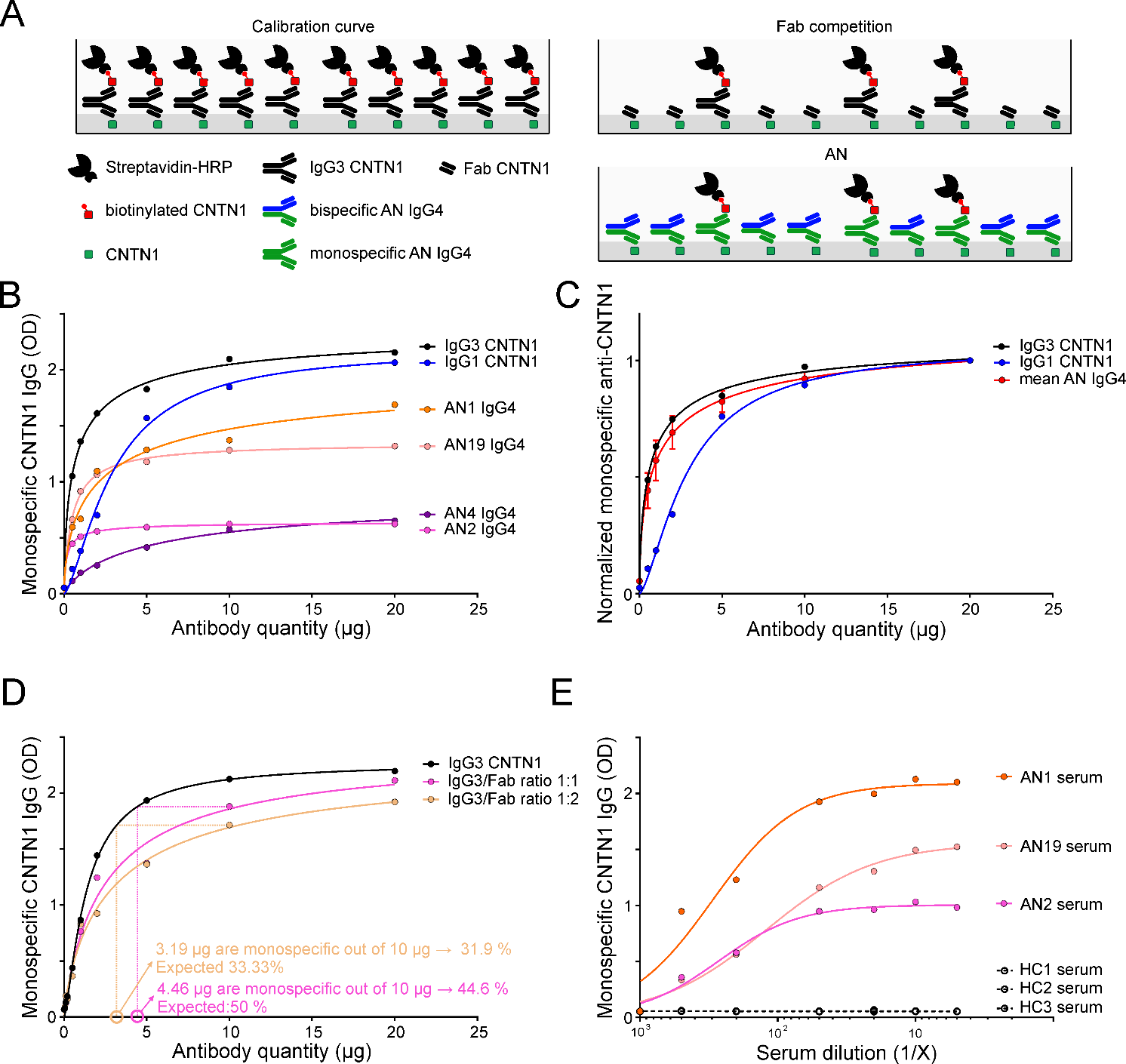


## Supplementary Figure 2 Method to evaluate the percentage of monospecific anti-CNTN1 IgG. (A) Schematic representation of the sandwich ELISA technique with examples of calibration curve, competition with Fab fragments, and tests of AN IgG4. For clarity, IgG are represented here with only one arm attached to the coated antigen to better represent the cross-linking antibodies, although IgG may also bind with both arms to the coated antigen. At saturating concentration, antibodies reacting to CNTN1 occupy every antigenic site. Competition with monovalent Fab proportionally decreases the fixation of cross-linking IgG (here a ratio of IgG3/Fab 1:2). In AN sera, both mono- and bispecific anti-CNTN1 IgG4 bind to CNTN1, but only the bivalent monospecific IgG4 cross-links CNTN1 to biotinylated CNTN1. Bispecific IgG4 behaves like monovalent Fab. (B) The levels of monospecific anti-CNTN1 IgG were measured by sandwich ELISA with increasing quantities of purified anti-CNTN1 IgG3 (black), IgG1 (blue), or IgG4 from four AN patients (AN1, AN, AN4 and AN19). Curve were fitted with a nonlinear regression using one site specific binding with Hill slope equation in GraphPad Prism. IgG3 and IgG1 induced the maximal cross-linking. Cross-linking increased proportionally to the quantity of IgG3 or IgG1, and reached a plateau around 10 µg. Purified IgG4 from AN patients also reached a plateau around 10 µg, but the maximal value varied between patients, indicating different levels of monospecific anti-CNTN1 IgG4. (C) The curves in B were normalized to the maximal OD at 20 µg, and the values from all AN patients were averaged. The binding profile of anti-CNTN1 IgG4 fitted more closely to that of anti-CNTN1 IgG3, than IgG1. (D) To validate the assay, anti-CNTN1 IgG3 were mixed with anti-CNTN1 Fab fragment (from purified IgG3) in 1:1 and 1:2 ratios to decrease the levels of monospecific IgG to 50% and 33.33% respectively. The OD values obtained with a quantity of 10 µg of IgG3/Fab mixture were interpolated to the anti-CNTN1 IgG3 calibration curve to calculate the corresponding quantity of monospecific IgG3. As expected, 1:1 IgG3/Fab mixture reduced the levels of monospecific IgG to 44.6 %, and 1:2 IgG3/Fab mixture to 31.9%. (E) The levels of monospecific anti-CNTN1 IgG were measured by sandwich ELISA in the serum of three AN patients (AN1, AN2, and AN19) and three healthy controls (HC1-3) at different serum dilutions. Like for purified antibodies, the levels of monospecific IgG in AN sera increased as serum dilution decreased, and reached a plateau for a dilution of 1/20.


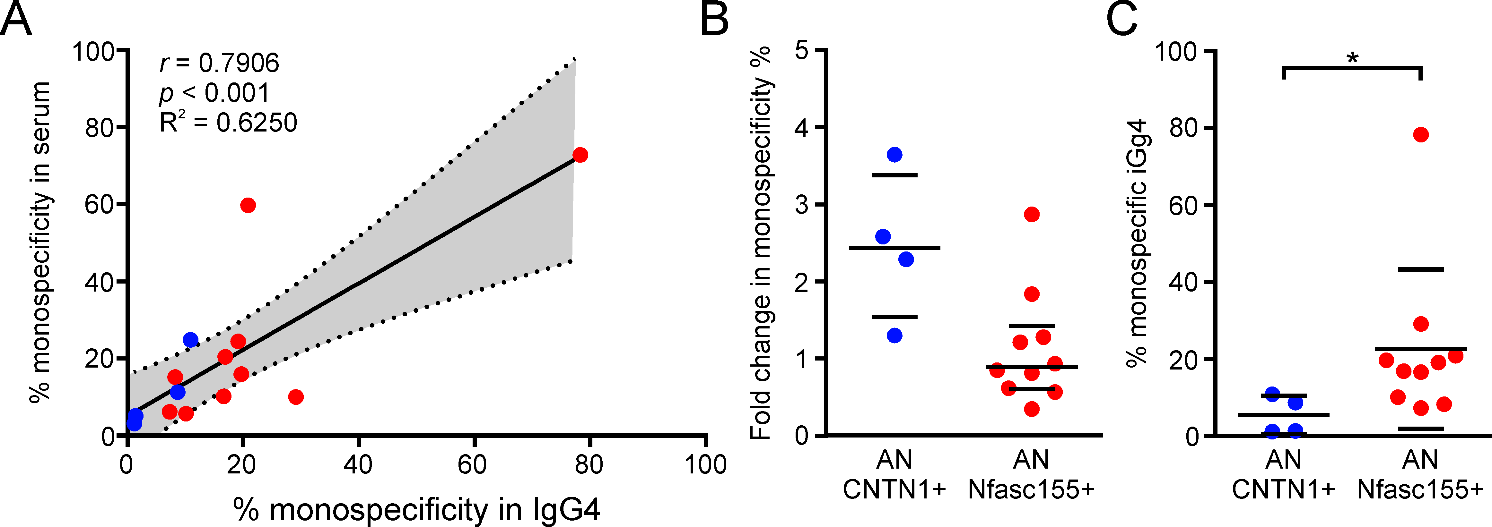


**Supplementary Figure 3** Correlation between percentage of monospecificity measured in sera and in purified IgG4 fraction. (A) The percentage of monospecific antibodies was measured in the serum and purified IgG4 fraction from AN patients with anti-CNTN1 IgG4 (n = 4; blue) or anti-Nfasc155 IgG4 (n = 10; red). The percentage of monospecific antibodies detected in serum strongly correlated with those found in the purified IgG4 fraction. P value, Spearman’s correlation coefficient (r), R square (R^2^) and 95% confidence band (dotted lines) are indicated on the graph. (B) The fold change in the percentage of monospecific antibodies between serum and IgG4 (% serum/% IgG4) were calculated in each patient. In CNTN1+ AN patients, the percentage of monospecific IgG appeared overestimated, but not in Nfasc155+ AN. Bars represent median and interquartile range. (C) The graph represents the percentage of monospecific anti-CNTN1 IgG4 or anti-Nfasc155 IgG4 detected in CNTN1+ AN and Nfasc155 + AN, respectively. Percentage of monospecific IgG4 were significantly higher in Nfasc155+ AN patients (* *P*<0.05 by unpaired two-tailed Student’s t tests).

##
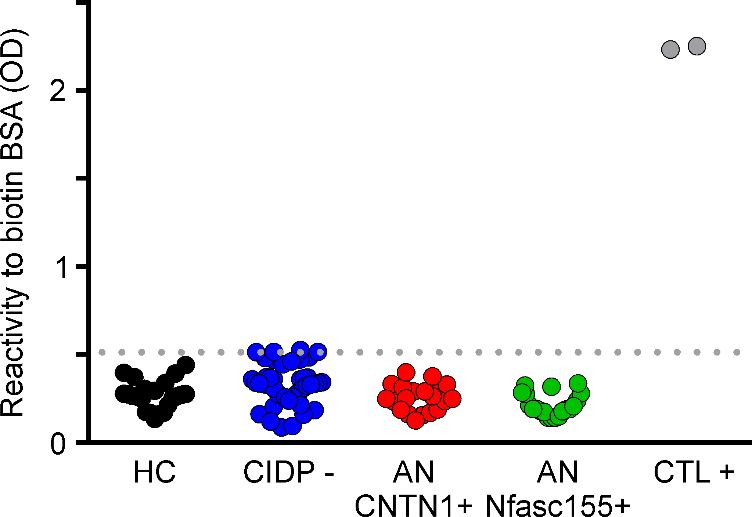


## Supplementary Figure 4 Absence of reactivity to biotin in CIDP and AN patients. The capacity of serum antibodies to bind to biotinylated bovine serum albumin (BSA) was measured by ELISA in healthy controls (HC; n = 23), seronegative CIDP patients (CIDP - ; n = 43), Nfasc155 + autoimmune nodopathy (AN Nfasc155+; n = 21) and CNTN1 + autoimmune nodopathy (AN CNTN1+; n = 20). ELISA plates were coated with 50 ng of biotinylated BSA, and incubated with the sera diluted at 1/50 dilution overnight at 4°C, and revealed with HRP conjugated goat anti-human IgG antibodies. As positive control (CTL +), streptavidin conjugated to HRP was used (n = 2). Sera were considered positive when the calculated absorbance was higher than the mean of HC + 3X SD. No reactivity to biotin was detected in CIDP or AN patients.


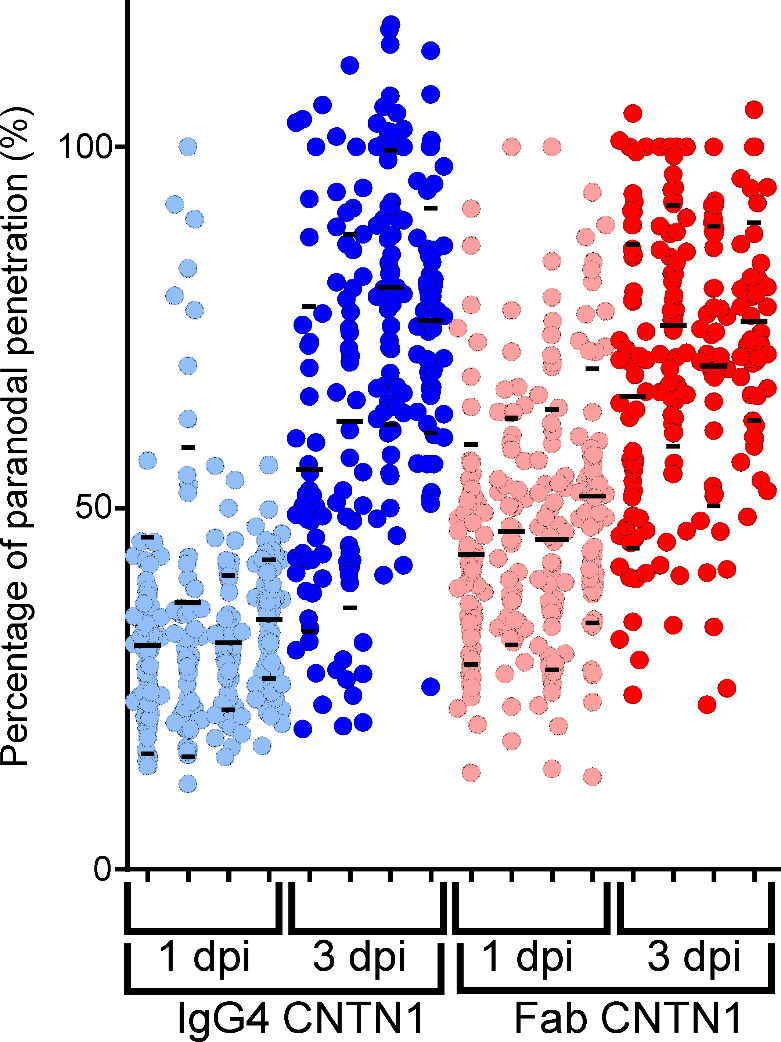


**Supplementary Figure 5** Monovalent Fab and native IgG4 penetrate the paranodal region. Adult Wistar rats have received a single intraneural injection of native CNTN1 reactive IgG4 (n = 4) or monovalent Fab (n = 4). Nerves have been collected 1 or 3 days post-injection (dpi) and the percentage of IgG infiltration was calculated in each animal at 1 and 3 dpi. The paranodal infiltration of native IgG4 reactive to CNTN1 or Fab were both higher at 3 dpi compared to 1 dpi. Bars represent mean and S.D.
